# Progressive left lower extremity weakness in a patient with multiple myeloma: A diagnostic dilemma

**DOI:** 10.1177/2050313X19833506

**Published:** 2019-03-05

**Authors:** Muhammad Sardar, Nasreen Shaikh, Saad Ullah Malik, Warda Faridi, Eli Balshan, Mihir Maniar

**Affiliations:** 1 Department of Medicine, Monmouth Medical Center, Long Branch, NJ, USA; 2 Department of Medicine, University of Arizona, Tucson, AZ, USA; 3 Department of Pathology, Monmouth Medical Center, Long Branch, NJ, USA

**Keywords:** Multiple myeloma, extramedullary plasmacytoma, left lower extremity weakness, metal artifact reduction protocol

## Abstract

Extramedullary plasmacytoma is a type of plasma cell dyscrasia that can present as solitary tumor or secondary to multiple myeloma. We experienced a case of intramuscular plasmacytoma in the left thigh muscles of a patient secondary to multiple myeloma. A 73-year-old male with relapsed multiple myeloma and bilateral hip arthroplasty complained of lxeft lower limb weakness and hip pain 3 months after relapse. He underwent contrast-enhanced magnetic resonance imaging of lumbar spine and hip which was inconclusive. Subsequently, patient had multiple admissions for progressive lower limb weakness. His clinical course was complicated by a biopsy-proven plasmacytoma of the neck. He received localized radiation therapy to the neck in addition to a change in multiple myeloma chemotherapy regimen, resulting in resolution of the neck mass but his left lower extremity weakness continued to worsen. Repeat magnetic resonance imaging of hip and spine revealed an intramuscular mass in left thigh which was consistent with the diagnosis of extramedullary plasmacytoma on biopsy. Localized radiation to the thigh accompanied with a change in chemotherapy improved his symptoms and a significant reduction in size of plasmacytoma was observed. When an unexplained lower limb weakness is encountered with a history of multiple myeloma, secondary intramuscular plasmacytoma should be considered.

## Introduction

Extramedullary plasmacytoma (EMP) is a neoplastic proliferation of plasmacytoid cells outside the medullary cavity.^1^ It can occur as solitary EMP or secondary to multiple myeloma (MM). The secondary spread of plasmacytoma at diagnosis of MM is not very common (up to 17%) and leads to poor prognosis.^2^ Intramuscular plasmacytoma (IMP) presents with localized swelling and pain because of mass effect.^3^ The mechanism of secondary spread includes local invasion from skeletal lesion after trauma or invasive surgical procedure or hematogenous spread.^4^ They are radiologically detectable and show two patterns of spread: diffuse muscle infiltration or intramuscular mass.^3,5^ We aim to describe a diagnostic dilemma of EMP presenting as lower limb weakness which remained radiologically undetectable until advance progression.

## Case presentation

**Figure 1.**
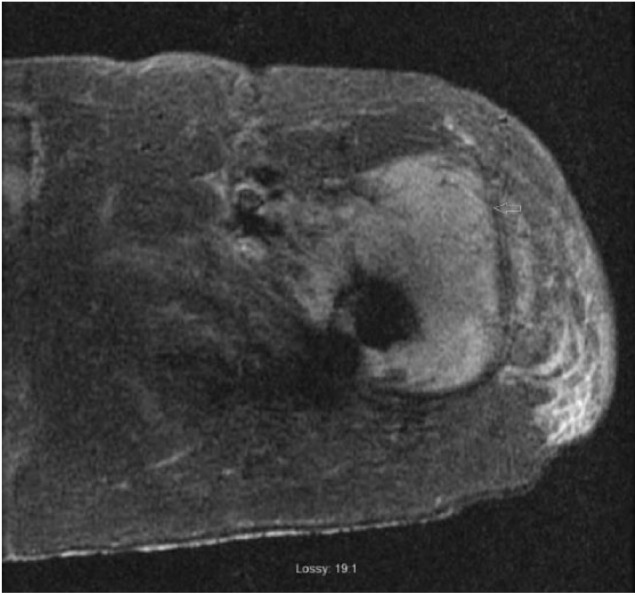
MRI of the hip region with metal artifact reduction protocol. A coronal STIR sequence image showing 7.7 cm × 5 cm hyper-intense mass adjacent to the greater trochanter of left femur.

**Figure 2.**
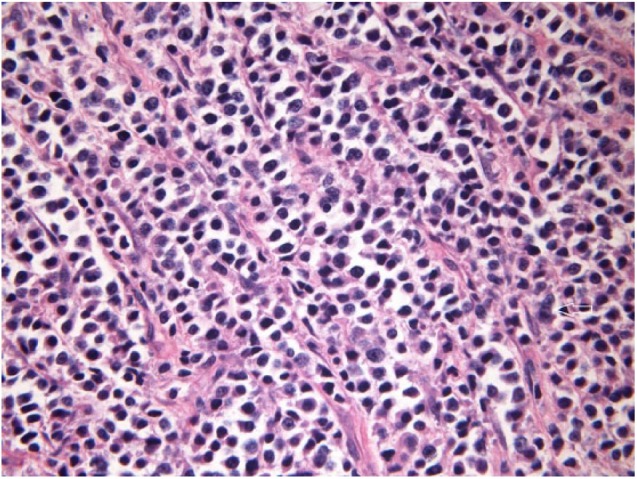
Muscle biopsy with H&E stain (40×) showing plasma cell neoplasm (white arrow).

**Figure 3.**
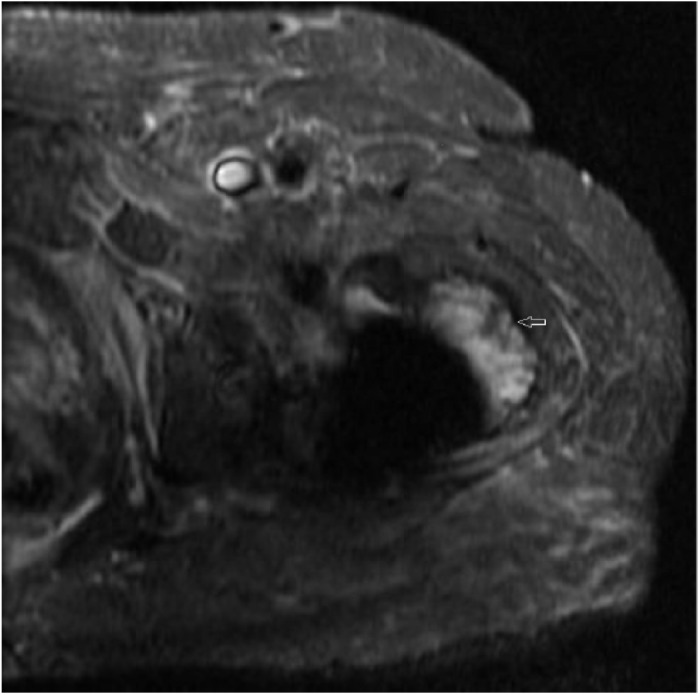
Contrast-enhanced axial T1-weighted MRI image of hip region showing a reduction is tumor mass (3.3 cm × 2.3 cm).

A 73-year-old gentleman with past medical history of left leg deep vein thrombosis (on apixaban 5 mg BID) and bilateral hip replacement 2 years ago was diagnosed with international staging system (ISS) stage 2 IgG kappa MM. Initial bone marrow biopsy revealed >20% plasma cells, whereas the fluorescence in situ hybridization (FISH) panel was positive for t(14-16) and negative for del13q14, t(4-14), t(11-14), t(14-20), P53 and hypodiploidy. Patient received four cycles of bortezomib and dexamethasone induction therapy but unfortunately had evidence of progressive disease as per international myeloma working group (IMWG) response categories. Patient’s regimen was switched to ixazomib, pomalidomide and dexamethasone and subsequently patient achieved partial response after third cycle. Meanwhile, patient presented to our hospital with slow onset dull pain localized to left hip along with lower extremity weakness of the same side. He was ambulatory without any complaint of urinary or stool incontinence. His vital signs were within normal limits. Neurological assessment of left limb revealed a decrease in muscle power while performing flexion and extension at hip and knee joint with a score of three by five and four by five, respectively. Rest of the physical examination was unremarkable. On admission, a T1- and T2-weighted contrast-enhanced magnetic resonance imaging (MRI) of hip and lumbar spine showed a stable heterogeneous enhancement in the sacrum consistent with patient’s known history of MM. Examination was limited because of susceptibility artifact from the metal prosthesis. Patient was later discharged with the advice of physical therapy. After 1 month, he was readmitted with a rapidly enlarging painless neck mass and progression of left leg weakness. Contrast-enhanced computed tomography (CT) scan of head and neck revealed a 7 cm × 10 cm × 3 cm mass encasing left carotid sheath. Ultrasound-guided biopsy showed CD138 positive plasmacytoid cells. He was switched to bortezomib, daratumumab and dexamethasone along with radiation therapy (50.2 Gy) for locoregional control. The differential diagnosis for his limb weakness included peripheral neuropathy secondary to MM, chemotherapy or an autoimmune process. Antiganglioside antibodies were ordered which came back negative. A trial of intravenous immunoglobulin also failed to relieve his symptoms. He was prescribed gabapentin (100 mg three times/day) for symptomatic relief and later discharged to a rehabilitation facility. In the next 3 months, there was complete resolution of neck mass on follow-up CT scan, but his lower extremity weakness worsened to a point that he could not walk. Repeat MRI of hip region with metal artifact reduction protocol revealed a 7.7 cm × 5.0 cm intramuscular mass abutting left hip prosthesis adjacent to greater trochanter (Figure 1). An ultrasound-guided core biopsy revealed small- to medium-size plasmacytoid cells with occasional plasmablastic cells. Immunohistochemistry positive for CD138 confirmed the presence of plasma cells (Figure 2). FISH reported strong kappa with no lambda immunoglobulin expression consistent with monoclonal B cells. Diagnosis of EMP secondary to MM was made. He was switched to elotuzumab, lenalidomide and dexamethasone accompanied with focal radiotherapy. After 4 weeks, his leg weakness improved along with significant reduction in tumor mass (3.3 cm × 2 cm) on follow-up MRI (Figure 3). Unfortunately, patient died due to aspiration pneumonia leading to hypoxic respiratory failure and sepsis.

## Discussion

MM is associated with neurologic complications mostly presenting as sensory weakness due to compression, infiltration or cytokine-mediated peripheral neuropathy.^6^ Our patient presented with pure motor weakness with no sensory involvement. This could be due to direct compressive effects of IMP or a MM-associated motor neuropathy. To the best of our knowledge, there is only one published case report on MM evolving into plasmacytoma of the rib and presenting with motor radiculo-neuropathy.^7^ EMP can occur in two ways: solitary plasmacytoma without prior involvement of bone marrow or secondary to pre-existing MM as in our case. IMP is a rare form of EMP that arises either as a de novo lesion or as extension from adjacent bone lesions.^5^ Direct extension is noted to be frequently preceded by bone trauma or an orthopedic surgical intervention. In our case, it extended from the adjacent bone most likely due to surgery for bilateral hip replacement. Most case reports describe it as appearing within 2 to 3 months of the surgical intervention.^8^ Our patient was diagnosed with extramedullary spread 2 years after his surgery. This may represent a late spread of myeloma triggered by surgical intervention. The mechanisms of extramedullary spread in MM are poorly understood. Proposed mechanisms of extramedullary spread include increased angiogenesis and decreased expression of adhesive molecules (VLA-4, CD-44 and CD 56) leading to faulty adherence of myeloma cells to bone marrow endothelium.^4,9^ Interestingly, our patient was negative for CD56 on initial flow cytometry. A retrospective study from Japan showed that t(14-16) was associated with lack of CD56 expression which was the case in our patient.^10^ Clinical presentation of extramedullary spread depends on the site and size of lesion. Surov et al.^5^ in the retrospective analysis of IMP patients observed that 55% presented with pain and swelling at the site of lesion, whereas rest of 45% were asymptomatic. On the contrary, our patient presented with weakness of left lower extremity and localized pain in his hip with no abnormal radiological findings for 5 months. IMP appears isointense on T1-weighted MRI and hyperintense on T2-weighted MRI compared to surrounding normal skeletal muscles and present either as intramuscular mass or diffuse infiltration.^5,11^ In our patient, IMP was undetected on the initial MRI but subsequent MRI detected presence of a large IMP. MRI with metal artifact reduction protocol is usually used to detect post-operative orthopedic complications in their early stages.^12^ Orthopedic hardware could interfere with imaging of soft tissue which may explain the delay in detection. Unfortunately, MM patients with IMP has poor prognosis. In a study by Varettoni et al.,^13^ extramedullary disease was associated with shorter overall survival (hazard ratio (HR) = 3.26, p < 0.0001) and progression-free survival (HR = 1.46, p = 0.04). Similarly, Wu et al.^14^ found that patients with extramedullary involvement had shorter overall survival when treated with chemotherapy as compared to patients with no extramedullary disease.

## Conclusion

This is a case of IMP with an unusual clinical presentation of pure motor weakness. Clinical symptoms can precede definite radiographic evidence of disease, so patients should be frequently monitored with serial imaging. Presence of metal prosthesis may interfere with interpretation of early sign of plasmacytoma, so in that case, an MRI with metal artifact reduction protocol or a positron emission tomography (PET)-CT should be employed instead. Our patient had high-risk MM, that is, t(14-16), which usually responds to proteasome inhibitors but our patient was initially refractory to bortezomib-based therapy and relapsed with a progression-free survival of 3 months on ixazomib-based regimen. EMP responded to local radiation therapy evident by resolution of neck mass and reduction in the size of left leg IMP.
